# Splice-Site Variants in the Gene Encoding GABA-A Receptor Delta Subunit Are Associated with Amphetamine Use in Patients under Methadone Maintenance Treatment

**DOI:** 10.3390/ijms24010721

**Published:** 2022-12-31

**Authors:** Yen-Feng Lin, Wen-Hai Chou, Tung-Hsia Liu, Chiu-Ping Fang, Hsiang-Wei Kuo, Po-Hsiu Kuo, Shih-Jen Tsai, Sheng-Chang Wang, Ren-Hua Chung, Hsiao-Hui Tsou, Andrew C. H. Chen, Yu-Li Liu

**Affiliations:** 1 Center for Neuropsychiatric Research, National Health Research Institutes, 35 Keyan Road, Zhunan Town, Miaoli County 350, Taiwan; 2 Department of Public Health & Institute of Epidemiology and Preventive Medicine, National Taiwan University, Taipei 100, Taiwan; 3 Department of Psychiatry, National Taiwan University Hospital, Taipei 100, Taiwan; 4 Department of Psychiatry, Taipei Veterans General Hospital, Taipei 112, Taiwan; 5 Institute of Population Health Sciences, National Health Research Institutes, Miaoli County 350, Taiwan; 6 Graduate Institute of Biostatistics, College of Public Health, China Medical University, Taichung 404, Taiwan; 7 Department of Psychiatry, Zucker Hillside Hospital, Northwell Health, Glen Oaks, New York, NY 11004, USA; 8 Department of Psychiatry, Donald and Barbara Zucker School of Medicine at Hofstra/Northwell, Hempstead, New York, NY 11549, USA; 9 Graduate Institute of Clinical Medical Science, China Medical University, Taichung 404, Taiwan

**Keywords:** amphetamine use, *GABRD*, methadone maintenance treatment, psychostimulant, polysubstance abuse

## Abstract

Chronic opioid use disorder patients often also use other substances such as amphetamines. The gene-based analysis method was applied in the genomic database obtained from our previous study with 343 methadone maintenance treatment (MMT) patients. We found that the gene encoding gamma-aminobutyric acid type A receptors (GABA-A receptor) delta subunit isoforms (*GABRD*) was associated with amphetamine use in heroin dependent patients under MMT in Taiwan. A total of 15% of the 343 MMT patients tested positive for amphetamine in the urine toxicology test. Two genetic variants in the *GABRD*, rs2889475 and rs2376805, were found to be associated with the positive urine amphetamine test. They are located in the exon 1 of the splice variant and altered amino acid compositions (T126I, C/T, for rs2889475, and R252Q, G/A, for rs2376805). The CC genotype carriers of rs2889475 showed a four times higher risk of amphetamine use than those with TT genotype. The GG genotype carriers of rs2376805 showed a three times higher risk of amphetamine use than the AA genotype carriers. To our knowledge, this is the first report that demonstrated an association of the delta splice variant isoform in the GABA-A receptor with an increased risk of amphetamine use in MMT patients. Our results suggest that rs2889475 and rs2376805 may be indicators for the functional role and risk of amphetamine use in MMT patients.

## 1. Introduction

Methadone maintenance treatment (MMT) is one of the standard pharmacological treatments for opioid use disorder (OUD) [1]. However, not all patients respond to this treatment. Approximately 54.7% of patients may still use heroin or other substances under MMT [2]. Polysubstance abuse is a known problem among opioid-dependent patients [3], including patients undergoing MMT [4]. Amphetamines (or methamphetamine) are one of the drugs that are frequently used by OUD patients in MMT program [5]. Amphetamine or methamphetamine has a chemical structure similar to the neurotransmitters dopamine and adrenaline with autonomic and psychomotor effects [6]. Methamphetamine can be metabolized into amphetamine through the liver cytochrome P-450 (CYP) isozyme 2D6 [7]. In other words, use of amphetamine or methamphetamine may exert similar psychoactive or autonomic effects for MMT patients. In our previous study on a candidate gene obtained from a large OUD genome-wide association analyses [8], amyloid beta (A4) precursor protein-binding family B member 2 (*APBB2*) gene has demonstrated an association with amphetamine use in OUD patients under MMT [9]. This indicated that OUD or MMT candidate genes may harbor candidates contributing to use of amphetamine use.

Alteration in the gamma-aminobutyric acid (GABA) system has been reported for reinforcement behaviors and intoxication in substance use disorder patients [10,11], and it plays a role in amphetamine use disorder [12]. GABA neurotransmitter binding to different GABA receptor subtypes may produce different responses (where the gamma-aminobutyric acid type A (GABA-A) receptor subtype produces chloride ionotropic effect, and gamma-aminobutyric acid type B (GABA-B) receptor subtype produces metabotropic effect). The GABA-A receptor structure contains five different protein subunits [13], while the GABA-B receptor structure contains two protein subunits [14]. At least 19 different genes encode these protein subunits and can be classified into eight families: α (1–6), β (1–3), γ (1–3), δ, ε, ρ (1–3), θ, andπ [15]. GABA-A receptor with γ-subunit (gene name *GABRG*) is located at the postsynaptic neuron and mediates phasic inhibition ionotropic effect, while that with δ-subunit (gene name *GABRD*) is located at the extra-synaptic neuron and mediates tonic inhibition response [16]. GABA-A α1 (GABRA1) and γ2 (GABRG2) have been reported to be associated with female amphetamine use disorder [17].

In this study, we reported a novel discovery of the GABA-A receptor δ subunit isoform (*GABRD*) as a candidate gene for combined use of amphetamine in the OUD patients under MMT. There has been abundant information regarding the genetic variants located in the exon region with missense mutations in several diseases [18]. This study demonstrated that two genetic variants as a result of missense mutations may play a functional role for amphetamine use in the patients under MMT. An alternatively spliced variant of *GABRD* may play a role in combined use of amphetamine in opioid dependent patients.

## 2. Results

### 2.1. The Characteristics of MMT Patients Who Used Amphetamine

A total of 51 of the 343 OUD patients (about 15%) under MMT tested positive for amphetamine in the urine toxicology (Table 1). These patients also had a higher percentage of morphine use than those patients tested negative for amphetamine (68.6% vs. 47.4%). There was no difference in treatment duration (weeks), benzodiazepine use, treatment adverse reactions in tachycardia/palpitations, and change in libido between the two groups.

### 2.2. GABRD Genetic Variants Were Associated with Amphetamine Use Tested by the Urine Toxicology

**Figure 1:**
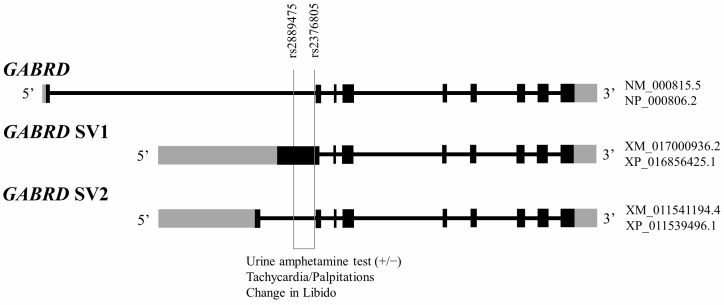
Three *GABRD* splice variants: *GABRD* (mRNA of NM_000815.5 and its encoding protein NP_000806.2), *GABRD* SV1 (splice variant 1, mRNA XM_017000936.2 and its encoding protein XP_016856425.1; containing the missense mutations), and *GABRD* SV2 (splice variant 2, mRNA XM_011541194.4 and its encoding protein XP_011539496.1) (GRCh38.p14), and the location of missense mutation SNPs in the *GABRD* gene structures. The black box indicates exon region and the gray box indicates untranslated region.

The *GABRD* gene is located at the chromosome 1p36.33 region with a spanning length of 11,413 bps, according to the chromosome version GRCh38.p14 (https://www.ncbi.nlm.nih.gov/gene/2563 (accessed on 12 August 2022)). Eleven single nucleotide polymorphisms (SNPs) were located within the genetic loci (Supplementary Table S1) with a high linkage disequilibrium (Supplementary Figure S1). Two SNPs, rs2889475 and rs2376805, located at the exon 1 region of *GABRD* splicing variant 1 (*GABRD* SV1, XM_017000936.2), which can be translated to predicated protein XP_016856425.1, were missense mutations (Figure 1). The reference C allele translated into threonine (Thr, or T) amino acid and the major T allele translated into isoleucine (Ile, or I) amino acid for rs2889475, whereas the reference G allele translated into arginine (Arg, or R) amino acid and the major A allele translated into glutamine (Gln, or Q) amino acid for rs2376805 (Supplementary Table S1). All of these 11 SNPs were significantly associated with the urine amphetamine test results of all MMT patients (Supplementary Table S2). In the missense mutation of the two SNPs, the reference (minor) genotype carriers had a 4.3 (for rs2889475) to 3.3 (for rs2376805) times higher percentage of amphetamine use (the positive results in the urine amphetamine test) than the major genotype carriers (Table 2). The heterozygous genotype had a 2.6 (for rs2889475) to 2.5 (for rs2376805) times higher percentage of amphetamine use than the major genotype carriers. The reference (minor) allele type carriers had about a 2 times higher percentage of amphetamine use than the major allele type carriers for both SNPs rs2889475 and rs2376805. These associations were mainly contributed from the MMT patients who tested positive in the urine morphine test (Supplementary Table S3).

We further calculated the mutation number as gene dose (GD) according to these two missense mutation carriers (Supplementary Table S4): the GD 0 represented the reference (minor) genotype carriers, GD 1 represented one or two mutated (major) alleles, and the GD 2 represented major genotype carriers. The GD was associated with the positive results in the urine amphetamine test from all MMT patients (*p* = 0.005) (Supplementary Table S5). GD 0 showed a 4.6 times higher percentage of amphetamine use than GD 2 (*p* = 0.021), where GD 1 showed a 2.5 times higher percentage of amphetamine use than GD 2 (*p* = 0.005). Similar statistically significant difference was also detected among the MMT patients who tested positive in the urine morphine test, but not in those who tested negative.

In *GABRD* rs2889475-rs2376805 haplotype association analyses, the haplotype frequencies were 0.77 for TA and 0.22 for CG haplotypes (Supplementary Table S6). The global haplotype was associated with the urine amphetamine test results (*p* = 0.013) in all MMT patients. The TA haplotype was negatively correlated with the positive results in the urine amphetamine test (*p* = 0.003), where the CG haplotype was positively correlated with the positive results in the urine amphetamine test (*p* = 0.001). These significant associations were observed in the MMT patients who tested positive in the urine morphine test.

### 2.3. No Significant Difference Was Found in the GABRD Genetic Variant Frequencies between the Age- and Gender- Matched Normal Controls and MMT Patients

To further study if the mutation variants of *GABRD* splicing variant 1 (SV1) contribute to the OUD, age- and gender-matched normal controls of 646 Taiwan subjects were compared to the 322 MMT patients in the genotype of two SNPs, rs2889475 (*p* = 0.668) and rs2376805 (*p* = 0.438) (Supplementary Table S7). No significant difference was found between the normal control subjects and the MMT patients. However, the urine amphetamine test positive of MMT patients were significantly different from controls (*p* = 0.002) in the genotype of the two SNPs. The results indicate the association of the two SNPs with amphetamine use but not opioid dependence.

### 2.4. GABRD Genetic Variants Were Associated with Methadone Treatment Adverse Reactions of Tachycardia/palpitation and Change in Libido

These two SNPs, rs2889475 and rs2376805, were also associated with some methadone maintenance treatment adverse reactions of tachycardia/palpitations and change in libido (Table 3). The major allele carriers who had a lower percentage of amphetamine use, had lower tachycardia/palpitation treatment emergent symptom scores (TESS) but bigger changes in libido than the reference allele carriers. Higher rating scores in the tachycardia/palpitations or change in the libido indicated more severe adverse effects. No significant associations were found between the *GABRD* genetic variants and the other clinical assessments.

### 2.5. The mRNA Isoforms Containing the Missense Mutation of GABRD Gene Were Measured in Different Human Brain Areas

To demonstrate the mRNA expression distribution profile of the three *GABRD* isoforms (Figure 1): *GABRD*, *GABRD* splicing variant 1 (SV1; containing the missense mutations), and *GABRD* splicing variant 2 (SV2), a quantitative real-time PCR was employed to measure the levels of their transcripts in the human brain regions of caudate nucleus, cerebellum, cerebral cortex, frontal lobe, hippocampus, insula, medulla oblongata, nucleus accumbens, substantia nigra, and temporal lobe. The *GABRD* SV2 showed the lowest expression level in comparison with the *GABRD* and the mutation containing *GABRD* SV1 in all brain regions. All three mRNA isoforms showed the highest levels of expression in the cerebellum brain region (Supplementary Figure S2).

## 3. Discussion

The role of the GABA receptor in polydrug use in patients with opioid use disorder (OUD) is not clear. Several reports have shown its involvement in amphetamine addiction [12]. This study is the first to report an association of δ subunit of GABA-A receptor (*GABRD*) with amphetamine use in opioid-dependent patients under MMT. There are three spliced variants of the GABRD subunit. Only one of the longest spliced variants (*GABRD* SV1) contains two SNPs (rs2889475 and rs2376805) of missense mutations in the exon 1 genetic locus in human. These two SNPs may be able to predict the severity of amphetamine use in the OUD patients under MMT. This discovery indicates a mechanism through a spliced variant of the δ subunit of GABA-A receptor for use of amphetamine or perhaps poly-substances in OUD patients under MMT.

Although δ subunit of GABA-A receptor have been reported to be involved with hypnosis, anxiolysis, and anticonvulsant [19], it is also noticed that the morphine may blunt the conditional place preference in *Gabrd*-knockout mice [20]. In other words, the *Gabrd* may be involved in mice depressive response after morphine treatment. It was hypothesized that if a δ subunit of GABA-A receptor antagonist was created, morphine or other opioids may produce antidepressant response [21]. The δ subunit of GABA-A receptor was also involved in the methamphetamine-induced locomotor activation and induction [20]. When the *Gabrd*-knockout mice treated with methamphetamine, the mice express normal sensitivity and normal locomotor activation and induction [20]. These observations suggest that the *GABRD* may contribute to a depressive response in OUD, which in turn explains in part why OUD patients “self-medicate” with amphetamine and other psychostimulants.

*Gabrd* was highly expressed in cerebellar granule cell layer [22], dentate gyrus granule cells of cerebellum [23], hippocampus [24], cerebral cortex, and thalamus [25] in the rat brain. Chronic amphetamine treatment may increase the response to endogenous norepinephrine of Purkinje neuron discharge in the rat cerebellum [26]. In this study, we found that the mRNA expression level of *GABRD* was higher in the human cerebellum region. Although it is not clear for the high expression level of *GABRD* in human cerebellum, a report from alcohol dependent patients showed that the GABRD protein levels in the cerebellum was decreased in patients due to the increased methylation promoter region of *GABRD* [27]. In a rat study, the *Gabrd* gene methylation upregulates the gene expression in nucleus accumbens involving in heroin-seeking behavior [28]. These results suggested that the regulation of *GABRD* expression in different brain regions may be essential for drug dependence.

This study also demonstrated that the brain *GABRD* had three RNA isoforms which are transcribed with the rank order of *GABRD* > *GABRD* SV1 > *GABRD* SV2 (Supplementary Figure S2). We attempted to measure the expression levels of the three isoforms in peripheral leukocytes, yet unfortunately the levels were too low to be detected. Therefore, it is difficult to determine the associations between the *GABRD* genetic variants and the expression of splice variants. However, T126I (rs2889475) mutation of major allele type may alter the polar amino acid Threonine (Thr, T) into nonpolar Isoleucine (Iso, I), and R252Q (rs2376805) mutation of major allele type may alter the basic (positively charged) polar Arginine (Arg, R) into acidic (negatively charged) polar amino acid Glutamine (Gln, Q) in *GABRD* SV1. Our results showed that the major mutant carriers had less tendency of combined amphetamine use than the minor mutant carriers in MMT patients. These two unique SNPs located in the exon 1 of *GABRD* SV1 may be indicators to predict amphetamine use in the MMT patients.

The *GABRD* gene was associated the MMT patients with positive urine amphetamine test in this study. A recent report showed a marginal association between *GABRD* haplotype and heroin dependence in 446 patients and 400 controls [29]. Their follow-up study reported no association between the *GABRD* genetic variants and methadone dose [30]. The present study further indicated the association of *GABRD* with OUD was mainly contributed from the MMT patients with a positive urine amphetamine test (Supplementary Table S7). *GABRD* SNPs (rs2889475 and rs2376805) were not associated with methadone dose, nor with treatment response determined by the urine morphine test results.

In the present study, the minor allele type (reference allele type) was a wild type, and the major allele type carriers on SNPs (rs2889475 and rs2376805) were mutants (Supplementary Table S1) for *GABRD* SV1. MMT patients with the minor allele had a higher percentage of amphetamine use than those with mutant (major) genotypes (Table 2). In other words, these mutant variants may prevent opioid users from using multiple drugs, such as amphetamine. The minor allele type carriers also had more severe side effects of tachycardia/palpitations yet smaller changes in libido than the major allele type carriers (Table 3). This results further validated that the minor allele carriers more likely to use amphetamine and may have more severe tachycardia/palpitations and smaller changes in libido.

In our previous study, we validated *APBB2* as a candidate gene for opioid-dependent as previous literature reported [8], using 344 Taiwanese patients from a genome-wide association study (GWAS) [9]. The *APBB2* genetic variants were associated with the urine amphetamine test results in MMT patients. However, the significant level (*p* = 0.0585) for the *APBB2* genetic variants was lower than that for the *GABRD* (*p* = 0.0014) when analyzed with the knowledge-based mining system for genome-wide genetic studies (KGG) for gene-based association analyses. Moreover, although both genes were associated with amphetamine use in patients under MMT, they were involved in different pathways. This implied that the behaviors of amphetamine use in MMT patients involved more than one pathway and single gene. Further studies should be performed to search for more potential targets in regulating the amphetamine use behaviors in MMT patients.

The limitation of this study included that the δ subunit of GABA-A receptor cannot be measured from peripheral lymphocytes. This limited further investigation of the role of the δ subunit expression in amphetamine use among the MMT patients. The protein products of the three mRNA isoforms were not investigated in the human cells or tissues in our study. Therefore, the function of mutant protein in *GABRD* SV1 warrants further investigations. Other limitations include use of categorical value for the urine morphine and amphetamine test results for analyses. The plasma methadone concentration was measured by high-performance liquid chromatography (HPLC) instead of the more precise liquid chromatography-mass spectrometry (LC-MS).

## 4. Materials and Methods

### 4.1. Subjects

All MMT patients received and signed the informed consent prior to participating in the study, which was approved by the institutional review boards of the National Health Research Institutes (EC0970504, Zhunan, Taiwan) and the seven participating hospitals [31]. The study was also registered with the National Institutes of Health (NIH) Clinical Trial (NCT01059747). 344 MMT patients were recruited according to the inclusion criteria: (1) 18 years of age and above, (2) receiving MMT for at least 3 months with regular attendance in the past 1 week, and (3) no more than 10 mg adjustment of the methadone dosage in the past 1 week. The exclusion criteria were pregnancy and co-morbidity requiring emergent treatment such as organic mental illness and schizophrenia. The details can also be found in the previous report [9].

In addition, 646 age- (±2 years) and gender- matched Taiwanese normal controls (data from the Taiwan Biobank) were included to compare with 322 MMT patients in this study. The use of data for normal controls was approved by the institutional review boards of National Taiwan University Hospital (201506095RINC).

### 4.2. Clinical Assessments

We obtained the demographics, clinical characteristics, and methadone treatment responses, including the treatment emergent symptom scores for the side effect evaluation from the medical records of the study subjects [31,32].

### 4.3. Urine Morphine and Amphetamine Screening

Urine specimens were collected prior to the administration of methadone, when the day of the clinical assessments were measured. The morphine and amphetamine screen tests were performed by a kinetic interaction of microparticles (KIMS) on an Integra 800 device (Roche Diagnostics, Basel, Switzerland) as described in the previous report [9]. It was considered positive when the urine morphine level is above 300 ng/mL or the urine amphetamine level is above 500 ng/mL. In our present analyses and previous reports [33,34,35,36], the urine morphine test was used as a surrogate measurement for the methadone treatment outcome.

### 4.4. Measurement of Plasma Methadone Concentration

Before the next methadone dose was given, 12 mL whole blood was collected from MMT patients. The whole blood was centrifugated at 2000× *g* in a Kubota 2800 centrifuge (Kubota Co., Osaka, Japan) for 20 min at 4 °C, and the supernatant as plasma was stored at −80 °C until use. The concentrations of racemic methadone in plasma were measured with high-performance liquid chromatography (HPLC) according to the method described in our previous report [35,37,38].

### 4.5. GABRD SNP Selection and Genotyping

Genomic DNAs of the 344 MMT patients were isolated from the buffy coat of the whole blood lymphocyte pellets using Gentra Puregene Blood kit (QIAGEN Sciences, Germantown, Maryland). The whole genome genotyping was operated by the Axiom Genome-Wide CHB 1 Array, which was population-optimized with a better coverage for the common alleles (MAF > 5%) in the Han Chinese Genome. The details of the quality control and the raw data can be assessed in the Gene Expression Omnibus (GEO accession number: GSE78098) [32]. The Michigan Imputation Server (https://imputationserver.sph.umich.edu (accessed on 1 June 2018)) imputed based on 1000G phase 3 v5 of reference panel, Eagle v2.3 of phasing, and EAS population. Using the genome-wide imputed database, we calculated the associations between the patients with positive vs. negative results in the urine amphetamine test to explore the candidate genes associated with amphetamine use in the MMT patients.

The candidate gene gamma-aminobutyric acid type A receptor δ subunit (*GABRD*) consists of 11 imputed SNPs resided within the *GABRD* gene locus fitted the profile of a coding RNA gene (Supplementary Table S1). One SNP (rs3121819) is located at the same position in the Axiom Genome-Wide CHB 1 Array and the genome-wide imputed database. More than one SNP significantly associated with the substance use disorder were reported in literature [29]. This gene was therefore selected for further statistical association analyses to explore its role in amphetamine use in the MMT patients.

To confirm the imputation results, a SNP rs2889475 was selected and genotyped using probe C_188870021_10 of TaqMan SNP genotyping assay (ThermoFisher Scientific, Waltham, MA, USA) and analyzed by the StepOnePlus Real-time PCR System (Applied Biosystems, Foster City, CA, USA) according to the manufacture’s protocol. One of the MMT patients was removed due to the transform imputation CC to genotyping result CT. Thus, a total of 343 samples were analyzed in this study.

### 4.6. GABRD Gene Isoform Expressions in Different Brain Region

Total RNAs from ten human brain regions, caudate nucleus, cerebellum, cerebral cortex, frontal lobe, hippocampus, insula, medulla oblongata, nucleus accumbens, substantia nigra, and temporal lobe, were purchased from Clontech (Clontech, Mountain View, CA, USA). 4 ug of RNA was converted to cDNA with random hexamer in 20 μL volume according to the manufacture of RevertAid H minus first strand cDNA synthesis kit (ThermoFisher Scientific, Waltham, MA, USA). The expression levels were analyzed with TaqMan probes of three *GABRD* isoforms (Supplementary Table S8 and Figure 1) and *TBP* (Hs00920497_m1, reference gene) using StepOnePlus real-time PCR system (ThermoFisher Scientific, Waltham, MA, USA). 200 ng cDNA, 20X TaqMan Probe, and 2X Gene expression master mix were mixed well in a 20 μL volume for each assay. The condition was starting at 95 °C for 10 min in the enzyme activation stage, flowing by 40 cycles of denaturation at 95 °C for 15 s, and annealing/extension at 60 °C for 1 min. The relative expression level of *GABRD* compared to that of TATA-box binding protein (*TBP*) endogenous control was defined as −ΔC_T_ = − [C_T, *GABRD*_ − C_T, *TBP*_], where C_T_ was the cycle threshold. The *GABRD* mRNA/*TBP* mRNA ratio was calculated from 2 −ΔC_T_ × K, in which K was a constant.

### 4.7. Statistical Analyses

Statistical analyses were performed by the SNP and Variation Suite, Version 8.4.0 (Golden Helix, Inc., Bozeman, MT, USA), SAS software, Version 9.4 (SAS Institute, Inc., Cary, NC, USA), PLINK Version 1.9 and Knowledge-based mining system for Genome-wide Genetic studies (KGG), Version 4.1. The demographics, divided by the results of the urine amphetamine tests, were analyzed using the Mann-Whitney U test for continuous variables and chi-square tests for categorical variables. The association analyses were calculated by the logistic regression analyses for the *GABRD* and treatment outcomes, withdrawal symptoms and side effect. General linear model (GLM) and logistic regression analyses were performed to further analyze SNPs in the exon regions, adjusted for urine morphine test. Considered the marginal significant benzodiazepine use in our patients, the logistic regression for *GABRD* and amphetamine use (+/−) in MMT was adjusted for the urine morphine test and benzodiazepine use. The power was calculated by the SAS PROC GLMPOWER procedure and the G-power tool for logistic regression. The haplotype association analyses by the results of the urine amphetamine tests were calculated using PHASE Version 2.1.1 with the generalized estimating equation (GEE) model for the logit link function based on the binomial distribution with the GENMOD procedure. The linkage disequilibrium (LD) plot was performed using HAPLOVIEW version 4.2.

## 5. Conclusions

In summary, we examined the genetic variants in the *GABRD*, a delta subunit of the GABA-A receptor, and found that two SNPs, rs2889475 and rs2376805, were associated with amphetamine use in a population of OUD patients under MMT. These two SNPs were associated with co-occurring stimulant use, specifically in amphetamine test positive urine, but not for addiction itself. The expression levels of three spliced variants of the *GABRD* gene in human brain were ranked in the following order: *GABRD* > *GABRD* SV1 > *GABRD* SV2. These two SNPs may result in amino acid changes in the *GABRD* SV1 splice variant. The increased number of mutations in the composition of these two SNPs was associated with the lower percentage of amphetamine use, the severity of change in libido, and tachycardia in MMT patients. In other words, the OUD patients with reduced number of mutations might have higher co-occurrence of polysubstance use, especially for amphetamine. These results suggest that the *GABRD* gene may be involved in the mechanisms underlying amphetamine use in opioid dependent patients who receive methadone maintenance treatment.

## Figures and Tables

**Table 1:** General characteristics of methadone maintenance treatment (MMT) patients grouped by the status of amphetamine use ^b^.

	Total		Urine Amphetamine Negative		Urine Amphetamine Positive		
Variable	*n*	Mean ± SD	*n*	Mean ± SD	*n*	Mean ± SD	*p*-Value ^m^
Age	343 ^a^	38.15 ± 7.70	289	38.12 ± 7.75	51	38.43 ± 7.59	0.68
Gender [Male (%)]	280	(81.63%)	236	(81.66%)	41	(80.39%)	0.82 ^c^
BMI, kg/m^2^	340	23.65 ± 3.52	286	23.60 ± 3.60	51	24.09 ± 3.01	0.16
Urine morphine (+)	173	(50.73%)	137	(47.40%)	35	(68.63%)	**0.005 ^c^**
Methadone dosage (mg/day)	343	55.27 ± 28.50	289	54.89 ± 28.68	51	58.53 ± 28.01	0.36
*R*-Methadone (ng/mL)	343	194.66 ± 123.67	289	196.57 ± 127.77	51	187.87 ± 100.25	0.94
*S*-Methadone (ng/mL)	343	142.61 ± 99.77	289	142.33 ± 102.24	51	147.14 ± 86.96	0.31
Addiction duration (years)	343	12.97 ± 7.51	289	12.96 ± 7.61	51	12.92 ± 7.16	0.95
Treatment duration (weeks)	341	64.83 ± 39.35	287	63.50 ± 37.69	51	71.59 ± 48.26	0.44
Benzodiazepine (Yes)	102	(29.74%)	81	(28.03%)	21	(41.18%)	0.06 ^c^
Treatment Emergent Symptoms Scale							
Tachycardia/Palpitations	25	1.44 ± 0.71	19	1.32 ± 0.58	5	2.00 ± 1.00	0.11
Change in Libido	103	1.77 ± 0.79	87	1.79 ± 0.79	15	1.67 ± 0.82	0.55

BMI, Body Mass Index. ^a^ one SNP genotyping result did not pass validation was removed from further analyses. ^b^ 3 patients had no urine amphetamine data. ^m^ Mann-Whitney U test. ^c^ Chi-square test. Bold font: *p*-Value < 0.05.

**Table 2:** Association analyses of *GABRD* and amphetamine use (+/−) in MMT patients.

		Urine Amphetamine Negative		Urine Amphetamine Positive					Urine Amphetamine Negative		Urine Amphetamine Positive			
SNP	Genotype	N^1^	(%)	N^1^	(%)	*p*-Value	FDR/ (Power)	Allele	N^2^	(%)	N^2^	(%)	*p*-Value	FDR/ (Power)
rs2889475														
	TT	182	(90.10%)	20	(9.90%)	**0.005**	**0.006**	T	463	(87.36%)	67	(12.64%)	**0.002**	**0.002**
	CT	99	(78.57%)	27	(21.43%)			C	115	(76.67%)	35	(23.33%)		
	CC	8	(66.67%)	4	(33.33%)									
	CC vs. TT	4.33 (1.16~16.20) ^odds^				**0.030**	(0.794)	C vs. T	2.13 (1.33~3.39) ^odds^				**0.002**	(0.913)
	CT vs. TT	2.56 (1.35~4.86) ^odds^				**0.004**	(0.934)							
rs2376805														
	AA	178	(89.90%)	20	(10.10%)	**0.010**	**0.011**	A	457	(87.21%)	67	(12.79%)	**0.004**	**0.004**
	AG	101	(78.91%)	27	(21.09%)			G	121	(77.56%)	35	(22.44%)		
	GG	10	(71.43%)	4	(28.57%)									
	GG vs. AA	3.32 (0.92~11.92) ^odds^				0.066	(0.690)	G vs. A	2.00 (1.25~3.18) ^odds^				**0.004**	(0.866)
	AG vs. AA	2.48 (1.31~4.72) ^odds^				**0.005**	(0.917)							

N^1^, subject number. N^2^, allelic number. ^odds^ Odds ratio (95% confidence interval). *p*-value, Logistic regression adjusted for urine morphine test (+/−) and benzodiazepine use. FDR, False Discovery Rate. Bold font: *p*-Value < 0.05.

**Table 3:** Association analyses of *GABRD* and the side effects in MMT patients.

Phenotype/SNP	Genotype	N^1^	Mean ± SD	*p*-Value	FDR/ (Power)	Allele	N^2^	Mean ± SD	*p*-Value	FDR/ (Power)
Tachycardia/Palpitations of side effect										
rs2889475	TT	14	0.98 ± 0.80	**0.0004**	**0.0005**	**T**	39	1.31 ± 0.88	**0.005**	**0.005**
	CT	11	1.86 ± 0.65		(0.799)	**C**	11	1.89 ± 0.70		(0.688)
	CC	0								
rs2376805	AA	13	0.99 ± 0.85	**0.003**	**0.003**	**A**	38	1.32 ± 0.90	**0.015**	**0.015**
	AG	12	1.79 ± 0.69		(0.720)	**G**	12	1.81 ± 0.72		(0.585)
	GG	0								
Change in Libido of side effect										
rs2889475	TT	60	2.02 ± 0.91	0.091	0.092	**T**	157	1.95 ± 0.93	**0.038**	**0.039**
	CT	37	1.78 ± 0.81		(0.507)	**C**	49	1.64 ± 0.79		(0.585)
	CC	6	1.22 ± 0.77							
rs2376805	AA	58	2.06 ± 0.89	0.091	0.092	**A**	155	1.96 ± 0.92	**0.018**	**0.039**
	AG	39	1.74 ± 0.81		(0.599)	**G**	51	1.61 ± 0.79		(0.690)
	GG	6	1.22 ± 0.76							

N^1^, subject number. N^2^, allelic number. Mean, adjusted for age and gender. SD, standard error × √n. *p*-Value, General linear model adjusted for urine morphine test (+/−). FDR, False Discovery Rate. Bold font: *p*-Value < 0.05.

## Data Availability

The data presented in this study are available on reasonable request from the corresponding author. The data are not publicly available due to privacy or ethical restrictions.

## Supplementary Materials

The following supporting information can be downloaded at: https://www.mdpi.com/article/10.3390/ijms24010721/s1.
