# Supplementary material for: Splice-Site Variants in the Gene Encoding GABA-A Receptor Delta Subunit Are Associated with Amphetamine Use in Patients under Methadone Maintenance Treatment
Source: Int J Mol Sci. 2022 Dec 31;24(1):721. doi: 10.3390/ijms24010721 (PMC9820820; doi:10.3390/ijms24010721)

Table S1. Single nucleotide polymorphisms in the *GABRD* coding region on chromosome 1.

SNP	Position	Location[a]	Function	HWP	MAF	Allele
rs3121819	2020388	5' UTR		0.199	0.22	G/A
rs28502222	2021166	5' UTR		0.199	0.22	C/T
rs28407473	2021171	5' UTR		0.199	0.22	C/T
rs3128318	2021343	5' UTR		0.199	0.22	A/C
rs3128321	2021813	5' UTR		0.199	0.22	C/T
rs3128322	2022997	5' UTR		0.199	0.22	A/G
rs3128323	2023641	5' UTR		0.199	0.22	A/G
rs2889475	2024545	**Exon1**	Missense / A**C**C ⇒ A**T**C, T [Thr] ⇒ I [Ile]	0.199	0.22	T/C
rs2376805	2024923	**Exon1**	Missense / C**G**G ⇒ C**A**G, R [Arg] ⇒ Q [Gln]	0.300	0.229	A/G
rs28574670	2027822	Intron4		0.250	0.217	G/A
rs28581504	2029487	Intron6		0.199	0.22	C/T

MAF = Minor allele frequency; HWP = *P*-value of Hardy-Weinberg equilibrium test.

[a] Localization: according to the isoform of *GABRD* SV1 mRNA (XM_017000936.2) at NCBI database GRCh38.p14.

Table S2. Association analyses of all *GABRD* SNPs and amphetamine (+/-) in patients under MMT

	Genotype		Allele	
SNP	*P*-value	FDR	*P*-value	FDR
rs3121819	**0.005**	**0.007**	**0.002**	**0.002**
rs28502222	**0.005**	**0.007**	**0.002**	**0.002**
rs28407473	**0.005**	**0.007**	**0.002**	**0.002**
rs3128318	**0.005**	**0.007**	**0.002**	**0.002**
rs3128321	**0.005**	**0.007**	**0.002**	**0.002**
rs3128322	**0.005**	**0.007**	**0.002**	**0.002**
rs3128323	**0.005**	**0.007**	**0.002**	**0.002**
**rs2889475 (Exon1)**	**0.005**	**0.007**	**0.002**	**0.002**
**rs2376805 (Exon1)**	**0.012**	**0.012**	**0.004**	**0.004**
rs28574670	**0.005**	**0.007**	**0.001**	**0.002**
rs28581504	**0.006**	**0.007**	**0.002**	**0.002**

P-value, Logistic regression adjusted for urine morphine test (+/-). FDR, False Discovery Rate.

Table S3. Association analyses of *GABRD* and amphetamine (+/-) in patients under MMT **with a positive urine morphine test**

SNP	Genotype	Amphetamine (-) N[1]	Amphetamine (-) (%)	Amphetamine (+) N[1]	Amphetamine (+) (%)	*P*-value	FDR	Allele	Amphetamine (-) N[2]	Amphetamine (-) (%)	Amphetamine (+) N[2]	Amphetamine (+) (%)	*P*-value	FDR
**rs2889475**														
	**TT**	89	(87.25 %)	13	(12.75 %)	**0.013**	**0.018**	**T**	221	(83.08 %)	45	(16.92 %)	**0.004**	**0.006**
	**CT**	43	(69.35 %)	19	(30.65 %)			**C**	53	(67.95 %)	25	(32.05 %)		
	**CC**	5	(62.50 %)	3	(37.50 %)									
	**CC vs TT**	4.11 (0.88~19.26) [odds]				0.073		**C vs T**	2.32 (1.31~4.11) [odds]				**0.004**	
	**CT vs TT**	3.03 (1.37~6.69) [odds]				**0.006**								
**rs2376805**														
	**AA**	87	(87.00 %)	13	(13.00 %)	**0.023**	**0.023**	**A**	217	(82.82 %)	45	(17.18 %)	**0.010**	**0.010**
	**AG**	43	(69.35 %)	19	(30.65 %)			**G**	57	(69.51 %)	25	(30.49 %)		
	**GG**	7	(70.00 %)	3	(30.00 %)									
	**GG vs AA**	2.87 (0.66~12.51) [odds]				0.161		**G vs A**	2.12 (1.20~3.74) [odds]				**0.010**	
	**AG vs AA**	2.96 (1.34~6.55) [odds]				**0.007**								

N[1], subject number. N[2], allelic number. [odds] Odds ratio (95% confidence interval)

P-value, Logistic regression. FDR, False Discovery Rate.

Table S4. *GABRD* gene dosage in the study population.

Gene Dose	N	Genotype rs2889475	Genotype rs2376805	Number of mutation
0	12	CC	GG	Wild type
1	2	C**T**	GG	1 mutation
	125	C**T**	**A**G	2 mutation
2	4	**TT**	**A**G	3 mutation
	200	**TT**	**AA**	4 mutation

rs2889475, Missense / A**C**C ⇒ A**T**C, T [Thr] ⇒ I [Ile]

rs2376805, Missense / C**G**G ⇒ C**A**G, R [Arg] ⇒ Q [Gln]

Table S5. Mutation number of gene dose association between *GABRD* and amphetamine (+/-) in patients under MMT.

Gene dosage	Genotype	Amphetamine **(-)** N	Amphetamine **(-)** (%)	Amphetamine **(+)** N	Amphetamine **(+)** (%)	***P*-value**
**Gene Dose in all MMT**						
	**0**	8	(66.67 %)	4	(33.33 %)	**0.005**
	**1**	99	(78.57 %)	27	(21.43 %)	
	**2**	182	(90.10 %)	20	(9.90 %)	
	**0 vs 2**	4.55 (1.26~16.46) [odds]				**0.021**
	**1 vs 2**	2.48 (1.33~4.65) [odds]				**0.005**
**Gene Dose in urine morphine test positive patients**						
	**0**	5	(62.50 %)	3	(37.50 %)	**0.013**
	**1**	43	(69.35 %)	19	(30.65 %)	
	**2**	89	(87.25 %)	13	(12.75 %)	
	**0 vs 2**	4.11 (0.88~19.26) [odds]				0.073
	**1 vs 2**	3.03 (1.37~6.69) [odds]				**0.006**

[odds] Odds ratio (95% confidence interval)

P-value, Logistic regression.

Table S6. Haplotype association analyses between *GABRD* and urine amphetamine test (+/-).

Block / Group	Haplotypes (Frequency)	Estimate	S.E.	Wald Chi-Square	*P*-value	Global *P*-value
**Block [rs2889475-rs2376805]**						
Overall patients	TA (0.77)	-0.75	0.25	8.96	**0.003**	**0.013**
	CG (0.22)	0.83	0.25	10.73	**0.001**	
Urine morphine test positive	TA (0.77)	-0.76	0.30	6.42	**0.011**	**0.026**
	CG (0.22)	0.89	0.31	8.16	**0.004**	

P-value calculated by generalized estimating equation (GEE) model for the logit link function based on the binomial distribution.

incorporating haplotypes frequency≧0.1 only.

Table S7. Association analyses of *GABRD* genotype frequency between MMT patients and the age- gender matched controls.

SNP	Genotype	NC		MMT patients		UAN/MMT patients		UAP/MMT patients		*P*-value (FDR)		
		N	(%)	N	(%)	N	(%)	N	(%)	*MMT vs. NC*	*UAN vs. NC*	*UAP vs. NC*
**rs2889475**										0.668	0.504	**0.002**
	**TT**	405	(62.7 %)	192	(59.6 %)	173	(63.6 %)	17	(36.2 %)	(0.767)	(0.554)	**(0.002)**
	**CT**	210	(32.5 %)	119	(37.0 %)	91	(33.5 %)	27	(57.4 %)			
	**CC**	31	(4.8 %)	11	(3.4 %)	8	(2.9 %)	3	(6.4 %)			
**rs2376805**										0.438	0.807	**0.002**
	**AA**	403	(62.4 %)	188	(58.4 %)	169	(62.1 %)	17	(36.2 %)	(0.767)	(0.807)	**(0.002)**
	**AG**	211	(32.7 %)	121	(37.6 %)	93	(34.2 %)	27	(57.4 %)			
	**GG**	32	(5.0 %)	13	(4.0 %)	10	(3.7 %)	3	(6.4 %)			

P-value, Logistic regression analysis. FDR, False Discovery Rate.

NC, Normal controls; MMT, Methadone maintenance treatment; UAN, Urine amphetamine negative; UAP, Urine amphetamine positive.

Table S8. The sequence of TaqMan probes and primers for GABRD expression assay.

Assay name	Accession number	Forward primer sequence	Reverse primer sequence	Probe sequence	Context sequence
GABRD	NM_000815.5	GCGCGCAGCAGCTC	GGTTGGAGCCCACGTAGTC	CCAGAGCGATGAATGAC	GCGCGCAGCAGCTCCGCGGCACCAG
GABRD SV1	XM_017000936.2	CTGCAAGGGCTCCCACAGT	GGTCAGACAGGCCAGGACTTAAT	CCCAGGTGCCCAGAA	NA
GABRD SV2	XM_011541194.4	GACCAATCCCCATCCTGTGT	CACGTAGTCGCCGATGTCAT	AGGAGGACAGAGCGAT	NA

Figure S1. The linkage disequilibrium (r^2) of the *GABRD* gene by Haploview 4.2. The black color indicates $r^2 \geqq 0$.

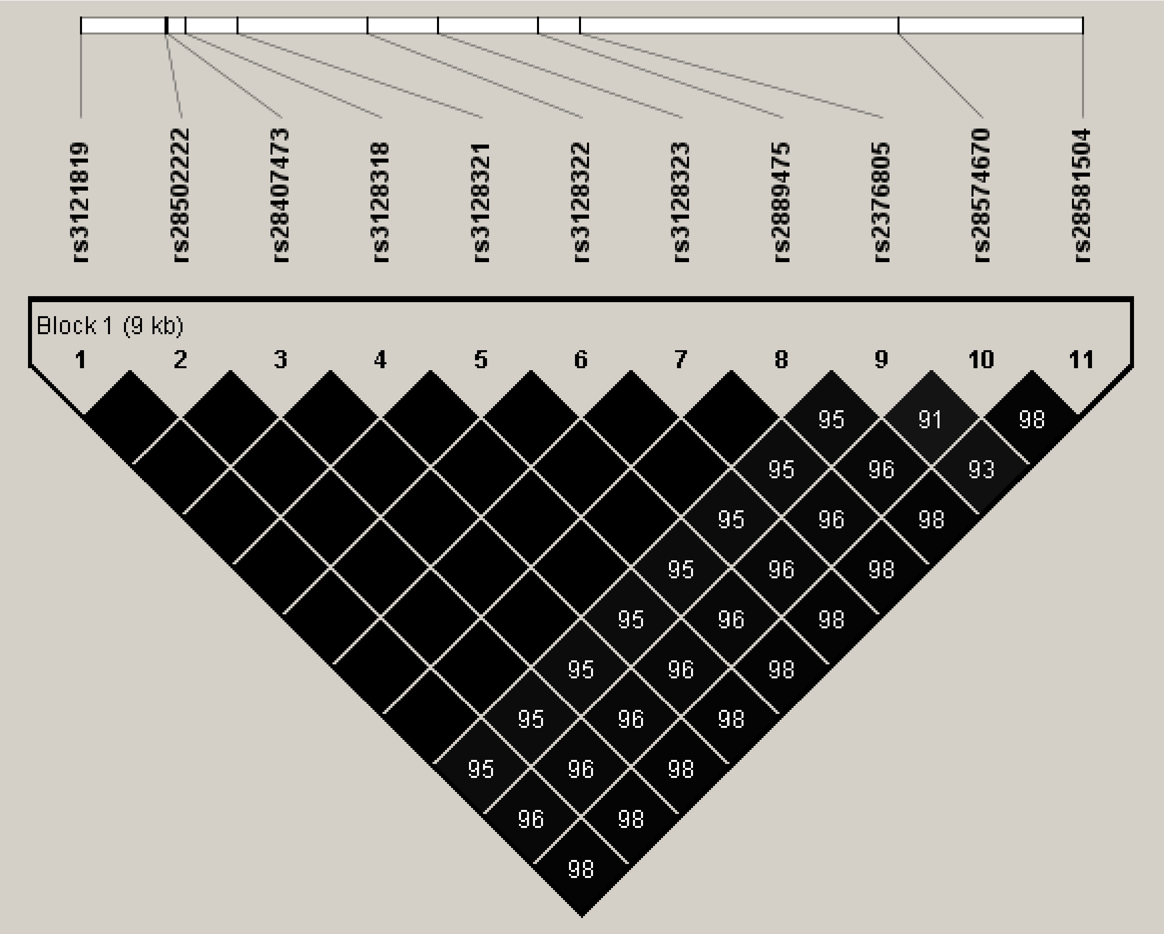

Figure S2. The expression of three GABRD isoforms in ten human brain regions. The GABRD SV1 protein contain the mutations of the two missense mutations of SNPs rs2889475 and rs2376805.

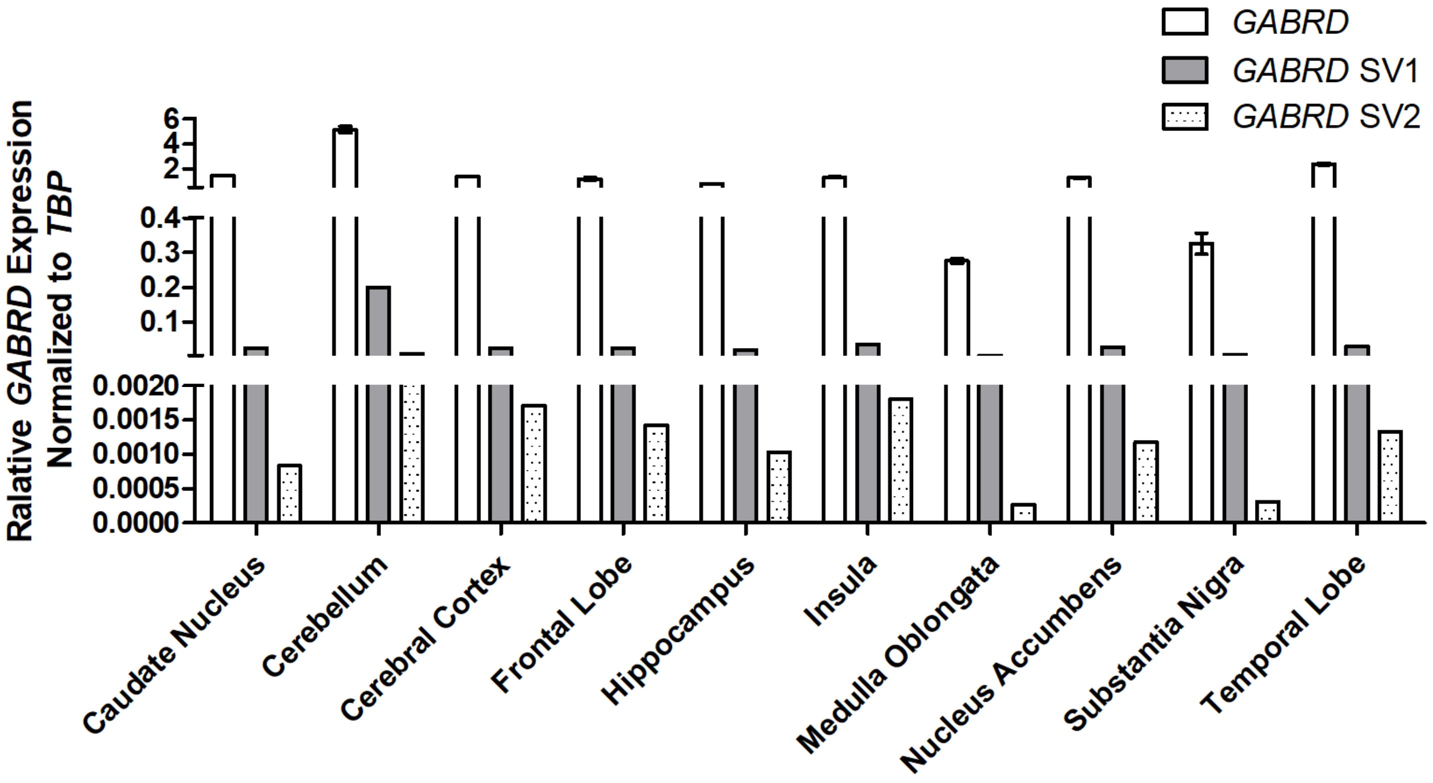

Supplement: Supplementary file 1 [file ijms-24-00721-s001.zip › ijms-1981914-supplementary.pdf]
